# The Medical Annual

**Published:** 1892-03

**Authors:** 


*The Medical Annual.* Pp. 652. Bristol: JOHN WRIGHT & CO. 1892.

The *Medical Annual* maintains its high level of usefulness and attractiveness. It is a more ambitious production than the *Year-Book*, inasmuch as its summaries have much more the character of special articles than of mere abstracts, and the bookbinder's work is better done. Among the more noteworthy of its articles are those by Mr. Hurry Fenwick on the Bladder, that on the Surgery of the Brain by Mr. Eve, that on the Surgery of the Spinal Cord by Mr. Thorburn, and that on Phthisis by Dr. Shingleton Smith. Specially acceptable are the original contributions of Dr. Armand Ruffer on Bacteriology, and of Mr. Pringle on Medical Photography.

The proof-reading of the *Annual* has not been very carefully done. We noticed "perityplitis" at p. 105 in Mr. Mayo Robson's note on Appendicitis, and "mesentry" in Plate VII. facing p. 547. The first two lines of p. 86 have surely escaped revision. Professor Horsley's name is distorted on p. 126. In the article on Phthisis, someone is responsible for having corrupted both the Christian name and surname of Dr. Heneage Gibbes, and on opposite pages Mr. Mayo Robson offers us a correct and an incorrect spelling of Dr. Einhorn's name. The advertisements in the *Annual* are encroaching too much on the other part of the book.

